# Comparative Study of Repertoire Classification Methods Reveals Data Efficiency of ***k***-mer Feature Extraction

**DOI:** 10.3389/fimmu.2022.797640

**Published:** 2022-07-20

**Authors:** Yotaro Katayama, Tetsuya J. Kobayashi

**Affiliations:** ^1^ Graduate School of Engineering, The University of Tokyo, Tokyo, Japan; ^2^ Institute of Industrial Science, The University of Tokyo, Tokyo, Japan

**Keywords:** TCR repertoire, repertoire classification, T-cell receptor, T-cell, k-mer, immunoinformatics, machine learning

## Abstract

The repertoire of T cell receptors encodes various types of immunological information. Machine learning is indispensable for decoding such information from repertoire datasets measured by next-generation sequencing (NGS). In particular, the classification of repertoires is the most basic task, which is relevant for a variety of scientific and clinical problems. Supported by the recent appearance of large datasets, efficient but data-expensive methods have been proposed. However, it is unclear whether they can work efficiently when the available sample size is severely restricted as in practical situations. In this study, we demonstrate that their performances can be impaired substantially below critical sample sizes. To complement this drawback, we propose MotifBoost, which exploits the information of short *k*-mer motifs of TCRs. MotifBoost can perform the classification as efficiently as a deep learning method on large datasets while providing more stable and reliable results on small datasets. We tested MotifBoost on the four small datasets which consist of various conditions such as Cytomegalovirus (CMV), HIV, *α*-chain, *β*-chain and it consistently preserved the stability. We also clarify that the robustness of MotifBoost can be attributed to the efficiency of *k*-mer motifs as representation features of repertoires. Finally, by comparing the predictions of these methods, we show that the whole sequence identity and sequence motifs encode partially different information and that a combination of such complementary information is necessary for further development of repertoire analysis.

## Introduction

T and B lymphocytes play a central role in the adaptive immunity of vertebrates, including human beings. Through the somatic recombination process called V(D)J recombination, T/B cells acquire diversities of T/B cell receptors (TCR/BCR) (1). These diversities are called the TCR/BCR repertoires. Clonal expansion of T/B cells, in response to infections of various pathogens, alters the repertoires (2). In particular, T cells are integral as the control center of the immune system to regulate other immune cells, including B cells. The development of next-generation sequencing (NGS) enables quantitative measurements of the somatically recombined regions of T cells’ genome, which encode the TCR, from cells collected from a wide range of tissues and conditions. NGS drives the progress of research on TCR repertoire from various aspects (3).

In basic immunology, public TCRs, T-cell receptors with identical or very close sequences shared across multiple individuals, have been studied intensively (4, 5). Before NGS, public TCRs were thought to be the result of multiple recombination events converging on the same amino acid sequences (6). However, recent studies based on NGS have revealed that the selection of antigen-specific or self-reactive TCRs may also contribute to the emergence of public TCRs (7–9). In applied immunology, quantitative measurements of T cell repertoire have already been employed for practical and clinical purposes. For example, the FDA (U.S. Food and Drug Administration) approved a test kit for micro residual disease, a type of leukemia (10).

On these backgrounds, the importance of bioinformatics and machine learning methods in processing and analyzing the sequenced repertoire data is increasing in both basic and applied immunology. For bioinformatics applications, several software tools(e.g., IMGT/HighV-QUEST (11), IgBLAST (12), MiXCR (13), etc.) have been developed to extract quantitative repertoire information from NGS data, and modeling of the dynamics of T cellrepertoire generation and selection is also being actively studied (14–17). For example, a mathematical model of recombination successfully classifies public and private TCRs (18).

For machine learning applications, repertoire classification tasks have been widely studied in the context of disease detection. As a result, various methods were proposed and have gradually evolved so as to exploit more complex information in the repertoire dataset: First, summary statistics of abundance distribution, such as Shannon’s entropy, were used for classifying and clustering the infection status and properties of repertoires. These statistics are scalar-valued and can be calculated only from the abundance distribution of sequences in a repertoire (19, 20). Similarly, distance-based methods were employed (21, 22). These methods classify or cluster repertoires based on distances between two repertoire distributions defined by metrics like the Morisita-Horn Similarity Index, which is frequently used in ecology. A new similarity index tailored to TCR has also been proposed (23).

These methods can be interpreted as unsupervised learning for the repertoire classification task, using only the abundance information of sequences and ignoring the sequence itself. Since these methods only use such summarized information, they can produce relatively robust results regardless of the number of samples. However, they abandon a large portion of potential information in the repertoire. They consider sequences as just independent labels regardless of their similarity. However, similar TCRs are experimentally suggested to behave similarly against antigens. Thus, analysis based on abundance alone inevitably has limitations. In addition, methods reducing a repertoire to a few parameters like those described above may not capture the complex mechanism of generation and maintenance of repertoires *in vivo*.

In order to address these problems, supervised learning frameworks have recently been employed and the increase of available repertoire datasets also boosts their development. Emerson et al. published the largest repertoire dataset (hereafter called “Emerson dataset”) at that time from 766 Cytomegalovirus (CMV)-infected and uninfected individuals (24). They employed the Fisher Exact Test to find the CMV-related subset of TCRs that appeared significantly more in the infected samples than in the non-infected ones. A binary classifier is then constructed, which uses the number of occurrences of the CMV-related TCRs in a given repertoire. Although this method also refers only to abundance information and discards sequence information, it achieves a high level of accuracy because the dataset is large enough to identify the significant fraction of TCRs. We call this method “the burden test” by following the preceding literature (25).

Natural language Processing (NLP) methods have also been applied to utilize receptor sequence information (26, 27). Among them, Widrich et al. (25) focused on the repertoire classification problem using one of the latest neural networks (NN) architectures which are popularly used in NLP. Repertoire data is essentially a collection of many short sequences for each subject (typically, about 10^5^-10^6^ sequences are obtained for each subject), and the repertoire classification problem is to assign a label to each of these collections. The number of the sequences being determinant of the label is few compared with the whole sequences in the repertoire. Therefore, it is essential to identify the determinant TCRs from a labeled training dataset. This kind of problem is called “Multiple Instance Learning” (MIL). In Widrich et al., NN is trained iteratively on small subsampled repertoires to predict the label of the original repertoire. The NN uses a technique called Attention to find the patterns of sequences associated with the repertoire label. Hereinafter, this method is referred to as “DeepRC.”

Both the burden test and DeepRC achieve good performance over the Emerson dataset of 766 subjects. However, the sample sizes in typical repertoire measurements are about an order of magnitude smaller than this dataset. In fact, according to TCRdb (28) as of April 2021, the largest database of repertoire sequencing data, 114 of 130 projects (88 %) have less than 100 samples (**Supplementary Table S1**). Whether these methods will work on datasets smaller than the Emerson dataset or not has yet to be tested. The burden test requires finding the TCRs observed significantly more frequently in the CMV positives than in the negatives *via* the Fisher Exact Test. When the sample size is small, it becomes difficult to find significant differences by such statistical tests. DeepRC employs a Transformer-like deep learning architecture, whose performance is also believed to depend significantly on the amount of available training data (29).

In this study, by investigating how these preceding methods behave in response to the change in the effective size of a dataset, we show that the performance of both methods deteriorates rapidly when the dataset size becomes smaller than a certain size. In order to compensate for the drawbacks of these methods, we also propose a new method (hereafter called “MotifBoost”) that works robustly on smaller datasets. For small to medium-sized datasets, a method is preferable to have a slow degradation in performance with respect to the decrease in data size. Additionally, if the method can achieve high performance comparable to the existing methods for sufficiently large datasets, it can be widely used regardless of the size of the datasets. We show that our proposed method satisfies both of these properties. MotifBoost adopts a *k*-mer based feature, which can exploit both sequence and abundance information without relying on strong but data-expensive representation learning conducted in deep learning (29, 30). We use Gradient Boosting Decision Tree (GBDT) as a classifier (31), because of its performance on small datasets (32, 33). We show that the performance of MotifBoost depends loosely on the dataset size and can achieve the comparative performance as DeepRC on the large Emerson dataset. We also compared MotifBoost with other previously proposed *k*-mer based methods, confirming that its performance is consistently superior to the other methods across the four different datasets. To further investigate why MotifBoost performs so well despite its simplicity, we visualized and examined the *k*-mer feature space. The result shows that repertoire classification is possible in the *k*-mer feature space at decent performance without any supervision, indicating that the conventional *k*-mer feature representation encodes and represents relevant information to the task. Finally, by scrutinizing the label predictions by all the three methods, we argue that there is a difference in the latent information of a repertoire employed between the burden test and either DeepRC or MotifBoost. This could hint at how we can integrate the best of those for further development.

This paper is organized as follows: In Materials and Methods, we provide an overview of our proposed method and the framework of the performance benchmark with two preceding methods. Then, in Results, we show how the performance of the three methods changes as the sample size changes. We also examine the stability of the performance with respect to variations in the training datasets. Next, we investigate the nature of the *k*-mer feature extraction to explain the low variance of the performance of MotifBoost. Finally, after mentioning a potential difference in the three methods, future directions are discussed.

## Materials and Methods

### MotifBoost

**Figure 1:**
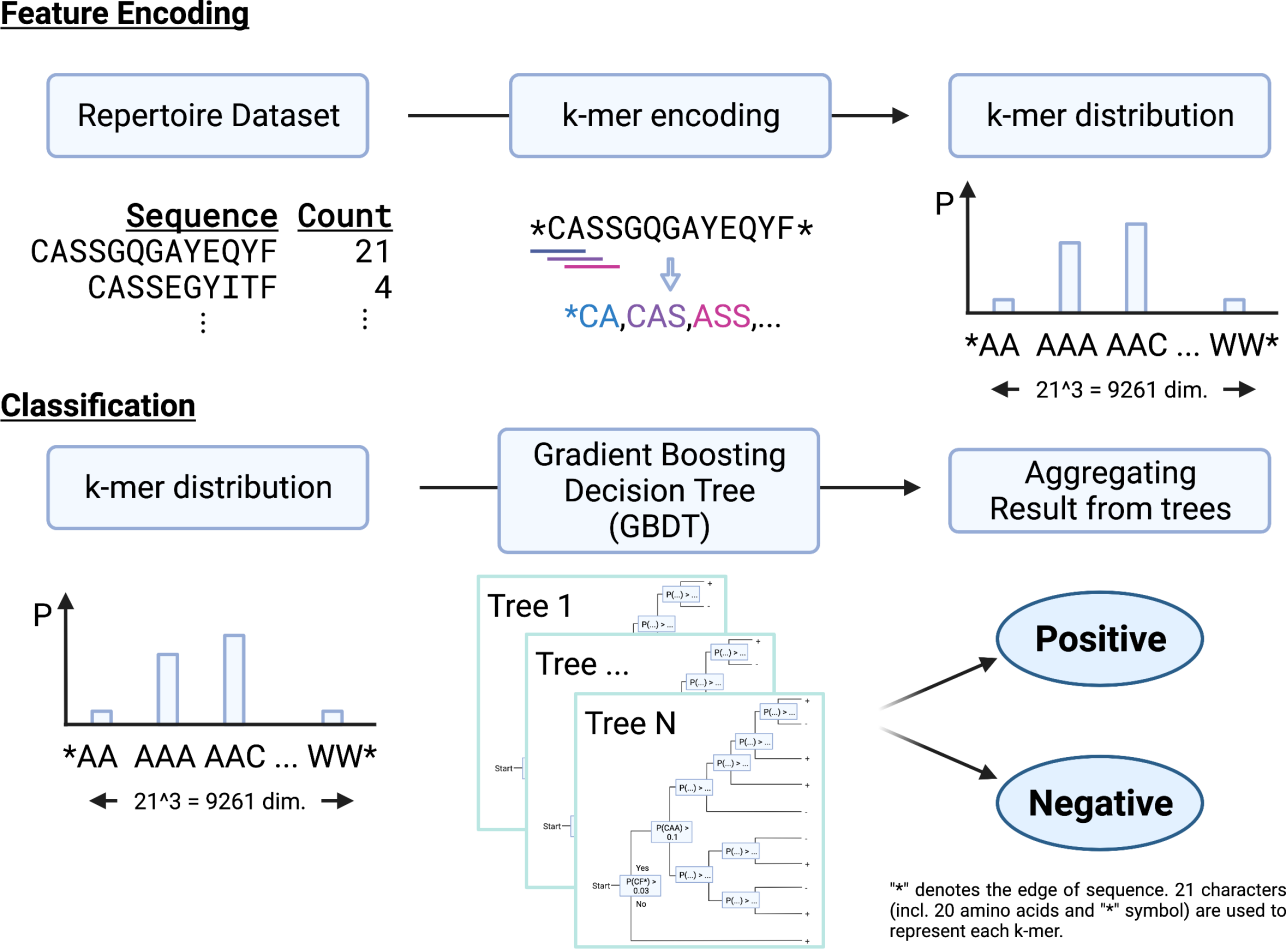
A schematic illustration of MotifBoost. MotifBoost employs k-mer distribution as a feature vector of a sample and GBDT as a classifier.

We propose a new repertoire classification method, MotifBoost, which is summarized in **Figure 1**. MotifBoost is inspired by the following two properties of TCRs. First, identical or similar TCRs may exhibit similar immune responses to antigens even across individuals. Various research supports this property. For example, even though TCRs are generated by the highly random V(D)J recombination process, there are public TCRs, a subset of TCRs with identical or very close sequences shared across multiple individuals (4, 5). It is reported that patients with the same infection history have such public TCRs in common (34). The success of the burden test, which uses the shared TCRs across individuals, also evidences the relevance of public TCRs to infections. Second, the response of TCRs to antigens is sometimes strongly influenced by “motifs,” short sequences of a few amino-acid lengths (35). One possible explanation for this property is that the presence of a particular motif affects the structure of the TCR antigen-binding site (36, 37).

Based on these observations, we employed the *k*-mer abundance distribution for the feature representation. All *k* consecutive amino acids in the sequences of a repertoire are listed to calculate their abundance distribution, which is to be used as the feature vector for the repertoire. Compared to the burden test, our approach treats a sequence as a set of motifs instead of a single sequence. This allows us to exploit sequence similarity information through the combinations of motifs. As our feature representation is a fixed-sized vector for a specific value of *k* regardless of the number of sequences or the sequence length, we can employ data-inexpensive models for classification, instead of complex deep learning architectures such as Transformer-like DeepRC (38). It should be noted that *k*-mer based approaches have been employed for the repertoire classification problem already. Sun et al. (39) adopted a sparse model (LPBoost) for the *k*-mer representation (*k*=3); Thomas et al. (40) employed *k-*mer representation and encoded each *k-*mer to feature vectors using Atchley factors (41), which represent the physicochemical properties of each amino acid. K-means clustering and SVM are combined to generate final predictions; Ostmeyer et al. (42) also formulated MIL by transforming the *k*-mer representation (*k=*4) into Atchley factors and performing linear regression and max-pooling operation on it.

As for the value of *k*, *k=*3 or *k=*4 has been widely used in previous studies like those mentioned above. In the case of *k=*4, the number of dimensions of the feature vector is about 160,000, which is the number of patterns of four consecutive residues composed of 20 human amino acids. This number is clearly too large for the repertoire classification task, as their sample size is 10^2^ at most. While Ostmeyer et al. adopted *k*=4, they also performed a dimensionality reduction. Every amino acid is represented as a five-dimensional biophysicochemical dense vector and any *k*-mer pattern is represented as a combination of those vectors. Therefore, we selected *k=*3 in this study. Each sample is represented by a multinomial distribution of *k*-mer abundance over 21^3^=9,261 dimensions, as we have 20 human amino acids and a symbol representing the edge.

The studies mentioned above have selected classification algorithms, which enable not only classification but also identification of important motifs. However, there is generally a tradeoff between interpretability and performance of methods. As we do not impose such a restriction in this study, we can adopt a more flexible algorithm. To achieve high classification performance, we chose GBDT (Gradient Boosting Decision Tree). GBDT is a kind of tree-based ensemble classifier that makes a final prediction by aggregating predictions of decision trees. It is much harder to interpret the output of tree-based models since they are ensembles of decision trees (31), but it can handle nonlinear correlations of motifs. Unlike other tree-based ensemble methods such as RandomForest, where decision trees are created independently, GBDT adds decision trees sequentially to minimize the error on training. GBDT is popularly used in data science competitions such as Kaggle (43). For its data efficiency is comparable to complex deep learning architectures in a considerable portion of tasks, it is widely used for tasks with limited data (32, 33). In addition, tree-based ensemble methods have been successfully utilized for relatively small size datasets in bioinformatics (44). For example, early gene expression analysis was conducted on 10^1^-10^2^ samples for 10^3^ genes to which tree-based ensemble methods have been employed (45). This sample size and number of dimensionality is very similar to those of the repertoire classification problem. This property is important because the repertoire classification problem is also severely data-limited as we saw earlier.

To improve the performance, we additionally employed the following techniques. First, we applied a data augmentation technique to increase the robustness of the model when it is trained on a small amount of data. Data augmentation is a common technique employed in data science to increase the effective amount of training data. For example, in the object detection task, we can crop, rotate, and reverse a picture to create similar pictures with the same object in it. In this study, we used sampling to create another repertoire from the existing one. Observed repertoire sequences from a subject can be seen as a sampling trial from the subject’s *in vivo* TCR distribution. By resampling the sequences from the observed data, we can simulate this sampling process and generate pseudo training data, which may contribute to the model’s ability to deal with the variance of the dataset. Second, hyperparameter tuning is performed, since the performance of GBDT is known to depend strongly on the hyperparameters. Hyperparameters are the parameters selected before training. In GBDT, for example, learning rate can be changed and we do not know the best value beforehand. Therefore, hyperparameter tuning is conducted by repeating the training for different values of the hyperparameters to see which set of values is best. In this process, only training data should be used. The details of the tuning are described later.

### Performance Measurement

We compare the performance of our proposed MotifBoost and two previously proposed supervised learning based methods, burden test and DeepRC. We use the Emerson dataset introduced earlier because the dataset is the one on which the latter two methods were validated and also because it is still one of the largest datasets being publicly available. To investigate the relationship between the dataset size and the performance of each method, we repeatedly sampled subsets of the dataset in different sizes and trained each method on each sampled subset. Then we performed a binary classification on the CMV infection status for each method. By following both papers of burden test and DeepRC, we measured the correctness of the classification result by ROC-AUC. This index is measured by the size of the Area Under the Curve (AUC) of Receiver Operator Characteristic (ROC) curve and used for evaluating a binary classifier, whose output is a scalar value. For such a classifier, we can determine a threshold and if a prediction score of a sample is above the threshold, the sample is predicted as positive by the classifier. ROC curve plots the False Positive Rate (FPR) for X-axis and True Positive Rate (TPR) for Y-axis by changing the threshold of the classifier. Therefore, the AUC of the ROC curve (ROC-AUC) is larger if the classifier has a lower FPR while retaining a higher TPR. The maximum ROC-AUC is 1.0, where the classifier can predict the scores of all the positive samples larger than those of all the negative samples. For such a classifier, we can set a threshold with which the classifier can predict all the samples correctly. Therefore, a larger ROC-AUC value is better.

The Emerson dataset consists of two cohorts, “Cohort 1” and “Cohort 2”, sampled in different medical facilities. They include 640 samples (CMV+: 289, CMV-: 351) and 120 samples (CMV+: 51, CMV-: 69), respectively. Cohort 1 in the original paper included 666 samples, but we excluded 25 samples with missing CMV infection status and one sample being unavailable in the published data.

In this study, Cohort 1 was used for training the models, and Cohort 2 was used for testing them. Hyperparameter tuning was also performed using only Cohort 1. This cohort-based train/test split is to avoid an undesired behavior called “shortcut learning,” in which a model learns to exploit undesirable information in data to predict the label (46). Because Cohorts 1 and 2 are sampled at different medical facilities, such undesirable information like batch effects may not be shared between them. Therefore, the possibility of “shortcut learning” is reduced under our setup compared to the mixed setup used in the original paper of DeepRC. Emerson et al. also employed the same setup as ours, and the setup is generally considered more appropriate for evaluating disease detection tasks than random train/test split of the mixed dataset (47).

By performing subsampling on this dataset, we can simulate small datasets. Hereinafter, repertoire sequence data from a single subject is referred to as a “sample,” and the entire 640 samples of Cohort 1 are referred to as the “full training dataset.” Subsampling is conducted as follows:

For a given dataset size *N*, we select *N* samples randomly without replacement from the 640 samples of the full training dataset. Because of no replacement, the subsampled dataset with *N=*640 is identical to the full training set. To maintain the comparability of the performance assessment, stratified sampling was performed so that the proportions of CMV positive/negative samples of subsampled datasets match that of the full training dataset as closely as possible. This is also a realistic setup. In the original training data, the proportions of positive and negative samples are controlled to be comparable. This level of control also can be expected even for other experimental situations with smaller sample sizes. A subset of the full training dataset generated by the above procedure is referred to as a “subsampled training dataset.”

Subsampled training datasets are created for N=25, 50, 100, 250, and 400. The performance of each method can depend on a certain choice of the subsampled samples, which mimics the situation that we happen to have a good or bad set of samples in an actual experiment. To evaluate the sample-dependent statistical variation of performance of the methods, for each *N*, we generated 50 independent subsampled training datasets. Then each method was statistically evaluated by measuring its performance with these 50 different subsampled datasets for each *N*. Training a method on one of the 50 subsampled datasets and measuring its ROC-AUC score is hereafter referred to as a “learning trial.” Thus, we performed 50 learning trials for each method and for each *N*.

All samples in Cohort 2 are used as the test dataset regardless of the training dataset size and of the classification method. All methods have no access to Cohort 2 samples during training.

To further validate the performance on the smaller datasets, the comparison is also performed on other datasets. We employed another CMV dataset (25 samples) from Huth et al. (48) and an HIV dataset (26 samples) from Heather et al. (20). These datasets are taken from the TCRdb (28) to satisfy the following conditions: the dataset has less than 100 samples; the dataset contains healthy samples (to avoid shortcut learning); the dataset is prepossessed by the authors (to validate the performance on various protocols). As these datasets consist of a single cohort, we employed cross-validation to test the performance.

Additionally, we compare Motifboost with two *k*-mer based repertoire classification methods introduced earlier to validate the superiority of the combination of the *k*-mer and GBDT. The first method is from Thomas et al. (40), which we call *k*-mer/SVM. The other method is from Ostmeyer et al. (42), which adopts linear regression based MIL. We call it *k*-mer/MIL. These methods are chosen by their popularity and the availability of implementation.

Detailed implementation and the parameters of each model are as follows: For MotifBoost, we employed LightGBM (49) as an implementation of GBDT and optimized its hyperparameters with the Bayesian optimization library Optuna (50). Optuna was run by its default parameters. The hyperparameter search was performed for each learning trial based on the cross-validated ROC-AUC score.

Data augmentation was also performed as follows: First, we randomly selected sequences from a sample with replacement to create an augmented sample. This is repeated until the number of sequences in the augmented sample becomes half of the original one. Note that the sampling probability for each sequence is weighted by its observation count to utilize abundance information. Second, this sampling was repeated five times for every training sample.

For the burden test, we implemented its algorithm by ourselves because the code is not available. The hyperparameter tuning is also performed as in the original paper, but we conducted a broader search than the original paper (**Supplementary Table S2**). The hyperparameter search was performed for each learning trial based on the cross-validated ROC-AUC score. The Fisher’s exact test is implemented based on SciPy and compiled by the JIT compiler library Numba for faster execution. The classifier is implemented based on immuneML (51).

For DeepRC, we adopted the author’s implementation and its default hyperparameters. In the original paper, the performance measurements were performed on a mixed Cohort dataset. We have patched the implementation so as to train it on Cohort 1 and test it on Cohort 2.

For *k*-mer/SVM, we employed the author’s code and parameters from the publication. For *k*-mer/MIL, we employed the implementation of immuneML. Although this method is sensitive to the parameters according to the original paper, its high computational cost did not allow us to conduct parameter search. Therefore, we adopted the best parameter set used in the publication.

Servers equipped with Intel CPU and operated by Ubuntu were used for numerical experiments. An NVIDIA RTX2080Ti GPU was used to run DeepRC. All experiments of MotifBoost, burden test, and the other *k*-mer based methods were conducted with Python 3.8.5, LightGBM 3.2.1.99, immuneML 1.2.1, Optuna 2.8.0, SciPy 1.6.2, NumPy 1.20.2, and Numba 0.50.1. Those of DeepRC were conducted with Python 3.6.9 and PyTorch 1.3.1, the same environment as that of the original paper.

### Visualization of the Feature Space

To investigate the feature space of MotifBoost, we employed an unsupervised dimensionality reduction algorithm called Gaussian Process Latent Variable Model (GPLVM) to visualize the feature vectors (52). GPy 1.9.9 (https://github.com/SheffieldML/GPy) was used to implement the model.

## Results

### The Performance of the Previously Proposed Methods Deteriorates Below Certain Sample Sizes

**Figure 2:**
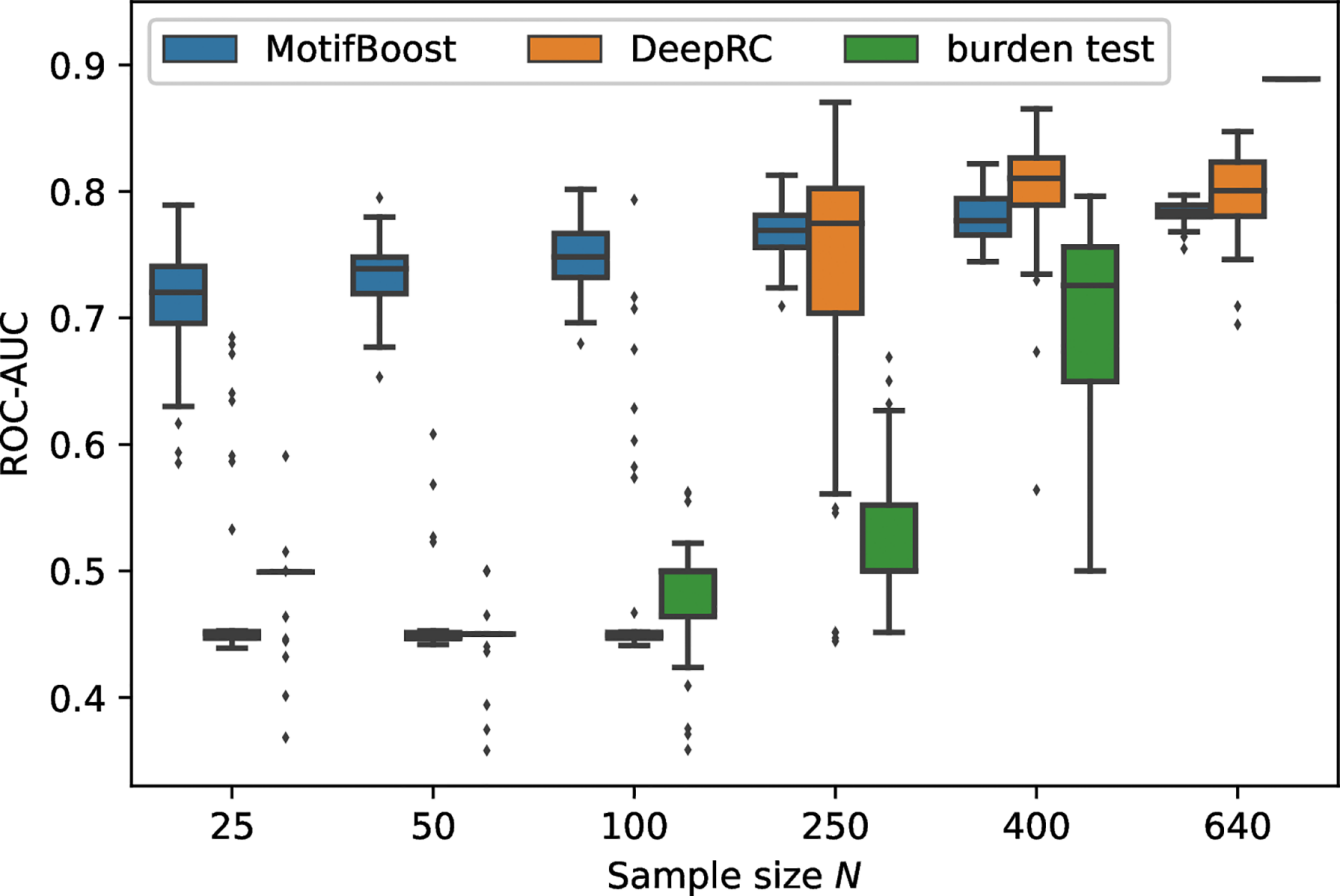
Performance change of each method in response to the change in the sample size of the training dataset. The box-and-whisker plot shows the median and lower and upper quartile of the ROC-AUC of each method. Note that the subsampling procedure is not performed for *N =*640. Thus, the variance for *N =*640 is not attributed to the randomness from subsampling. It is derived from the randomness of the methods themselves such as the choice of initial values. As the burden test is deterministic, its score for *N =*640 is shown as a bar without any variance.

We measured the classification performance of the three methods by ROC-AUC score with varying *N*, the number of training samples (**Figure 2**).

Being trained on the full training dataset (640 samples), the burden test achieved the best performance with the mean ROC-AUC score of 0.889 (as we will see later, the burden test is deterministic, therefore we do not show confidence intervals.). On the other hand, the mean ROC-AUC score of DeepRC is 0.80 ± 0.03 and that of MotifBoost is 0.78 ± 0.01. As the sample size *N* was reduced to *N*=400, the mean ROC-AUC score of the burden test decreased with a large variance of the scores among learning trials. When *N*=250 or less, the burden test can no longer learn. The performance of DeepRC was maintained even when the sample size *N* was reduced to 400. However, the same instability and rapid deterioration of performance were observed when *N=*250. DeepRC also cannot learn at *N*=100 or less.

### MotifBoost Performs Better Than the Other Methods at Small Sample Sizes

Trained on the full training dataset, MotifBoost achieved the equivalent level of the mean ROC-AUC score to DeepRC, with a difference of only 0.012, which falls within the confidence interval of DeepRC’s score. The performance of MotifBoost declines slowly as the sample size *N* decreases (**Figure 2**). However, the performance degrades only by 0.069 even if *N* is reduced to 25, i.e., the sample size is reduced by 96 % from the full training dataset (640 samples). When the sample size is 640 or 400, DeepRC shows slightly higher performance on average than MotifBoost. However, as discussed below, DeepRC has a large variance in performance. The mean performance difference between MotifBoost and DeepRC falls within this variation. The average performance of the three methods can be summarized as follows: Trained on the full training dataset (640 samples), the burden test shows the best performance, and the DeepRC and MotifBoost work equivalently. When the sample size is 400, they outperform the burden test because of its catastrophic breakdown. When the sample size is reduced further below 250, only MotifBoost could maintain the performance stably.

This result is also consistent with those on three additional datasets with small sample sizes (*N=*25 or *N=*26) listed in **Table 1**. They include one dataset from CMV patients and two datasets from HIV patients, each of which corresponds to *α* and *β* chain repertoires. We also create a dataset with *N*=25 from the Emerson data by subsampling. On all four datasets, MotifBoost shows better performance than other methods.

**Table 1:** Comparison of the mean ROC-AUC score on additional small datasets among all methods.

Condition (Chain, Sample Size)/Source	MotifBoost	DeepRC	burden test	*k*-mer/SVM	*k*-mer/MIL
HIV (*α*, *N*=26) /Heather et al.	**1.00**	0.57	0.51	0.99	0.58
HIV (*β*, *N*=26) /Heather et al.	**1.00**	0.50	0.38	0.93	0.93
CMV (*β*, *N*=25) /Huth et al.	**0.93**	0.35	0.90	0.63	0.47
CMV (*β*, *N*=25(subsampled)) /Emerson et al.	**0.71**	0.48	0.49	0.51	0.59

The first three datasets are from Heather et al. (20) and Huth et al. (48), the former of which includes repertoires of α and β chains. The last dataset is created from the Emerson dataset. For the datasets from Heather et al. and Huth et al., 5-fold CV was conducted three times. For the dataset from Emerson et al., the same setup used in **Figure 2** at N = 25 was used. Therefore, the models are trained on the Cohort 1 dataset and the scores are measured on the Cohort 2 dataset 50 times. The best results are shown in bold.

In addition, the experiment of CMV (*β, N=*25)/Emerson et al. of **Table 1** is trained and tested on the different datasets (Cohort 1 and 2). This means that the performance of MotifBoost on small datasets stays high even if the training and test datasets are different. It is important to evaluate the method’s ability to mitigate the batch effects. To check the ability, we further conducted two experiments (**Table 2**). We employed the datasets of Emerson et al. and Huth et al., both of which are studying human T-cell receptor *β*-chain with CMV infection. First, the ROC-AUC score of all methods was measured using the Emerson et al. (Cohort 2) dataset as the training dataset and the Huth et al. dataset as the test dataset. Then, the role of the datasets were swapped, and the same measurement was conducted. The results show that the ROC-AUC score of MotifBoost is consistently high compared to the other methods. Out of five methods, the ROC-AUC score of MotifBoost is tied for first when trained on the Emerson et al. dataset and is 2nd place when trained on the Huth et al. dataset. On the other hand, the performance of DeepRC is volatile. DeepRC is ranked first in the ROC-AUC score when trained on the Huth et al. dataset but fifth when trained on the Emerson et al. dataset. The burden test and *k*-mer/MIL do not perform well in either experiment. *k*-mer/SVM performs as well as MotifBoost when trained on the Emerson et al. dataset. However, when trained on Huth et al. dataset, its performance is below that of DeepRC and MotifBoost.

**Table 2:** Comparison of the mean ROC-AUC score of all methods trained and tested on different datasets.

Training data	Test data	MotifBoost	DeepRC	burden test	*k*-mer/SVM	*k*-mer/MIL
Emerson et al.	Huth et al.	**0.66**	0.38	0.53	**0.66**	0.53
(*N*= 120)	(*N*= 25)					
Huth et al.	Emerson et al.	0.72	**0.82**	0.45	0.63	0.51
(*N*= 25)	(*N*= 120)					

Datasets of Cohort 2 of Emerson et al. and Huth et al. for human T cell receptor β-chain with the CMV infection status are emplyed. One dataset is used as the training dataset, and the other is used as the test dataset. The models were trained to distinguish the CMV infection status. Two experiments were conducted by swapping the role of datasets. Each method was trained three times for each experiment, and the mean ROC-AUC score was measured. The best result for each experiment is shown in bold.

Nevertheless, the difference in the performance between the experiment of **Table 1** and the experiment of **Table 2** demonstrates that the batch effect still affects the methods. The methods that scored high performance in the former experiment, such as MotifBoost and burden test, drop the score in the latter experiment. However, MotifBoost still keeps the highest score among all methods. Therefore, MotifBoost can be evaluated as relatively immune to the batch effect.

To further validate the superiority of MotifBoost to other *k*-mer based methods, we compared it with *k*-mer/SVM and *k*-mer/MIL. All methods were trained on the full dataset (N=640), and their performances were measured in the same way as **Figure 2**. MotifBoost again shows better performance than the others in this setting (**Table 3**). For the datasets with small sample sizes, this trend was invariant as in **Table 1**.

**Table 3:** Comparison of the mean ROC-AUC score on the large datasets among three *k*-mer based methods.

Condition (Chain, Sample Size)/Source	MotifBoost	*k*-mer/SVM	*k*-mer/MIL
CMV (*β*, ***N*** =640)/Emerson et al.	**0.78**	0.52	0.71

The same setup as in **Figure 2** at N=640 was used. Therefore, the score of MotifBoost was taken from **Figure 2**. k-mer/SVM and k-mer/MIL were trained on the full training dataset and the scores were measured on Cohort2 dataset three times. The best result is shown in bold.

Moreover, MotifBoost can perform stably even if the number of sequences involved in a repertoire is small. In **Table 4**, we tested MotifBoost and burden test on the dataset created by trimming the sequences included in each sample of the Emerson data (*N*=640). MotifBoost can still keep its performance at 0.1% of the number of sequences of the Emerson dataset. For such a lower number of sequences, the burden test reduces its performance more greatly.

**Table 4:** Comparison of the mean ROC-AUC score among all methods trained on datasets with different average numbers of sequences in each sample.

Average number of sequences					
(Sampling ratio)	MotifBoost	DeepRC	burden test	*k*-mer/SVM	*k*-mer/MIL
3.9 x 10^5^ (10%)	**0.80**	0.71	0.79	0.55	0.72
3.9 x 10^4^ (1%)	**0.80**	0.71	0.67	0.55	0.73
3.9 x 10^3^ (0.1%)	**0.78**	0.71	0.61	0.51	0.67

The same setup as in **Figure 2** at N=640 for the Emerson dataset was used, except the average number of sequences in the training dataset (Cohort 1) was decreased by subsampling. The average number of sequences in the test dataset (Cohort 2) was 3.0×10^5^, and no subsampling was performed on it. The number in parenthesis denotes the sampling ratio. All methods were trained on the three independently subsampled datasets for each sampling ratio. The best results are shown in bold.

Computationally, MotifBoost requires less powerful hardware than the other methods. DeepRC uses deep learning and requires dedicated hardware such as GPUs. The burden test repeats the computationally expensive Fisher exact test, which is further burdened by the hyperparameter search. It also has to store the counts of all sequences in all the samples, which consumes a bigger RAM space in a naive implementation, but an efficient implementation has not yet been available. In our implementation and Python environment, DeepRC on GPU took 1.5 hours to train the full training dataset; the burden test with parameter search on CPU took about six hours using about 100GB of memory; *k*-mer/SVM also took about six hours; *k*-mer/MIL took more than a week. By contrast, MotifBoost on CPU took about three hours using about 50GB of memory. Note that MotifBoost can be further accelerated by using GPUs.

### MotifBoost Gives Reproducible Results for Different Datasets if its Size is Comparable

In **Figure 2**, the average performance of MotifBoost gradually increases with the increase in the number of samples, which is accompanied by a steady decrease in the performance variance. This property manifests the stability of learning. In contrast, for the other methods, the performance jumps abruptly at certain sample size, at which the variance also increases significantly. In addition, DeepRC shows a greater variance than MotifBoost in performance even beyond the critical sample size. This suggests that the results of the burden test and DeepRC can vary depending on the differences of the samples involved in the training dataset or on the stochastic nature of the method, especially near the critical sample size.

**Figure 3:**
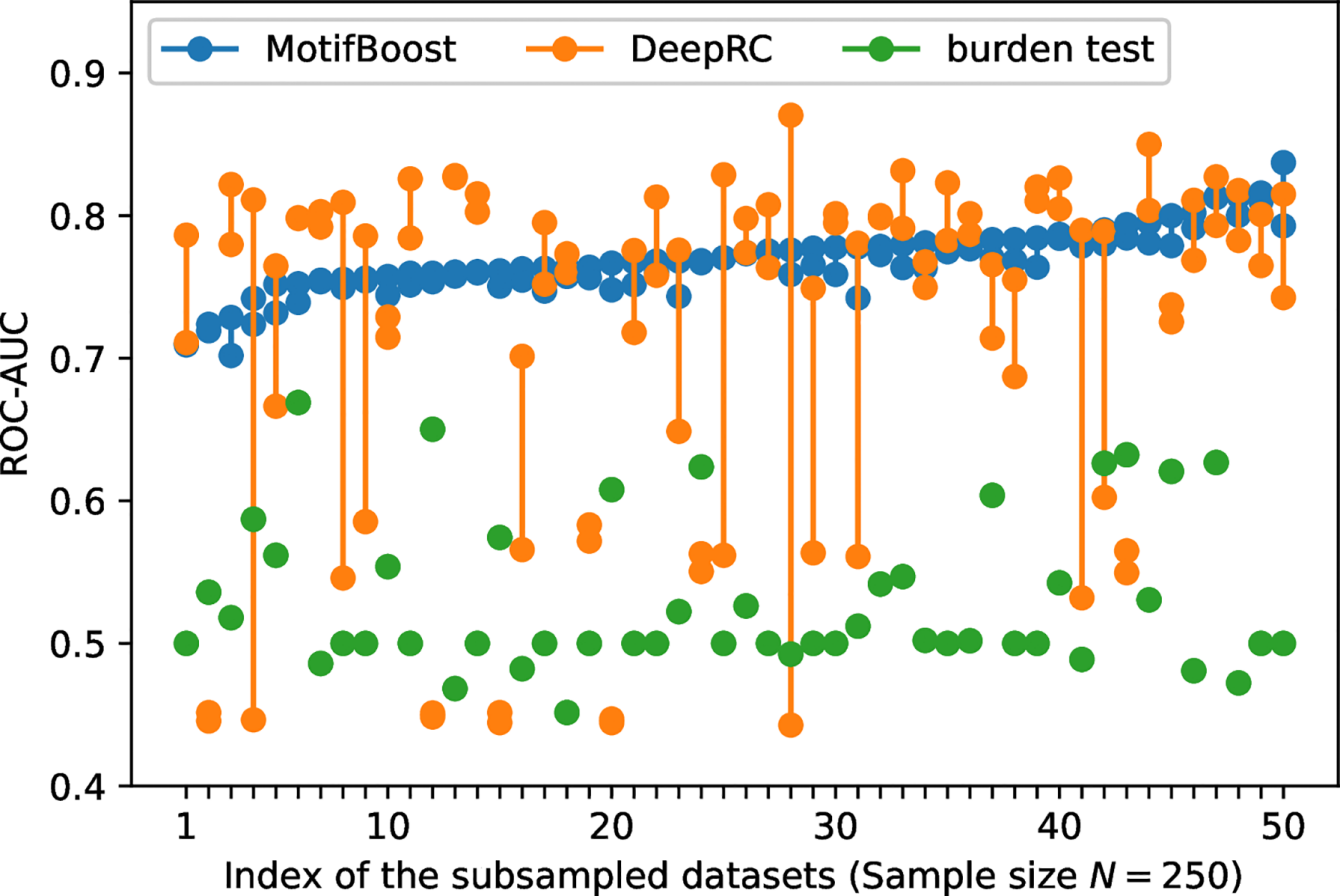
The variation of the ROC-AUC score between two learning trials trained on each subsampled dataset. The ROC-AUC scores of the two trials (circles) are plotted against the index of the 50 subsampled datasets. The index is sorted by the maximum ROC-AUC score of MotifBoost.

To further investigate the sources of variances, for each dataset used for the first trial, we conducted the second learning trial on the same training dataset. For this experiment, we chose the subsampled training datasets of 250 samples on which the variance of DeepRC was the largest (**Figure 2**). We performed the second learning trial of each method on each of the 50 subsampled datasets (The first learning trials are those shown in **Figure 2**). Then we compared the results of the first and the second learning trials of each subsampled dataset as shown in **Figure 3**.

The burden test showed no variation in the ROC-AUC score between the two learning trials because its algorithm is almost deterministic except for the choice of the initial value of the Newton method. To choose the initial value, we employed a commonly used algorithm, the method of moments, in which the initial value is deterministically obtained based on the average and variance of the training samples. Because a pair of the first and second learning trials use the same subsampled dataset (one out of the 50 subsampled sets), the burden test is completely deterministic in this study. This indicates that, for the burden test, the large variation of the ROC-AUC score at *N*=400 in **Figure 2** is exclusively attributed to the difference of samples involved in each subsampled training dataset.

In contrast, **Figure 3** shows that the performance of DeepRC can vary greatly compared to the other methods between the learning trials even if being trained on the same subsampled training dataset. The training process of DeepRC includes repeated random samplings of sequences in the training samples. The variability of performance indicated in **Figure 3** is due to this stochastic nature of DeepRC.

In addition, we found that the ROC-AUC scores of DeepRC are almost always low for some samples, which suggests the sample-dependent variation of performance. To confirm that, we performed another three learning trials on the four subsampled training datasets for which the ROC-AUC score of DeepRC was less than 0.6 in both the first and the second learning trials. For the two samples, the ROC-AUC score was less than 0.5 five times in a row. This implies that even though the size of the datasets is the same, the performance of DeepRC can also vary greatly, like the burden test, due to the difference of samples involved in the training dataset.

The potential instability of learning, originating from either sample dependence or stochasticity of training, is not desirable for practical use because it hampers us to derive a statistically confident conclusion from data, especially when the prediction from the methods cannot be validated in some other way (we could spot the instability in this investigation because the test dataset is labeled by CMV infection, but this is not the case in the usual situation of infection prediction). Compared with the other two methods, MotifBoost is also stochastic as it employs data augmentation and GBDT, but it balances high performance and small variance between trials (**Figure 3**).

In addition, the performance is also less sensitive to the differences of samples in the dataset (**Figure 2**), and it achieves the maximum ROC-AUC score of over 0.7 for any subsampled training dataset. Thus, MotifBoost has desirable reproducibility and stability to all the variations from samples, training processes, and the size of samples.

### Strong Feature Extraction of *k*-mer Representation

**Figure 4:**
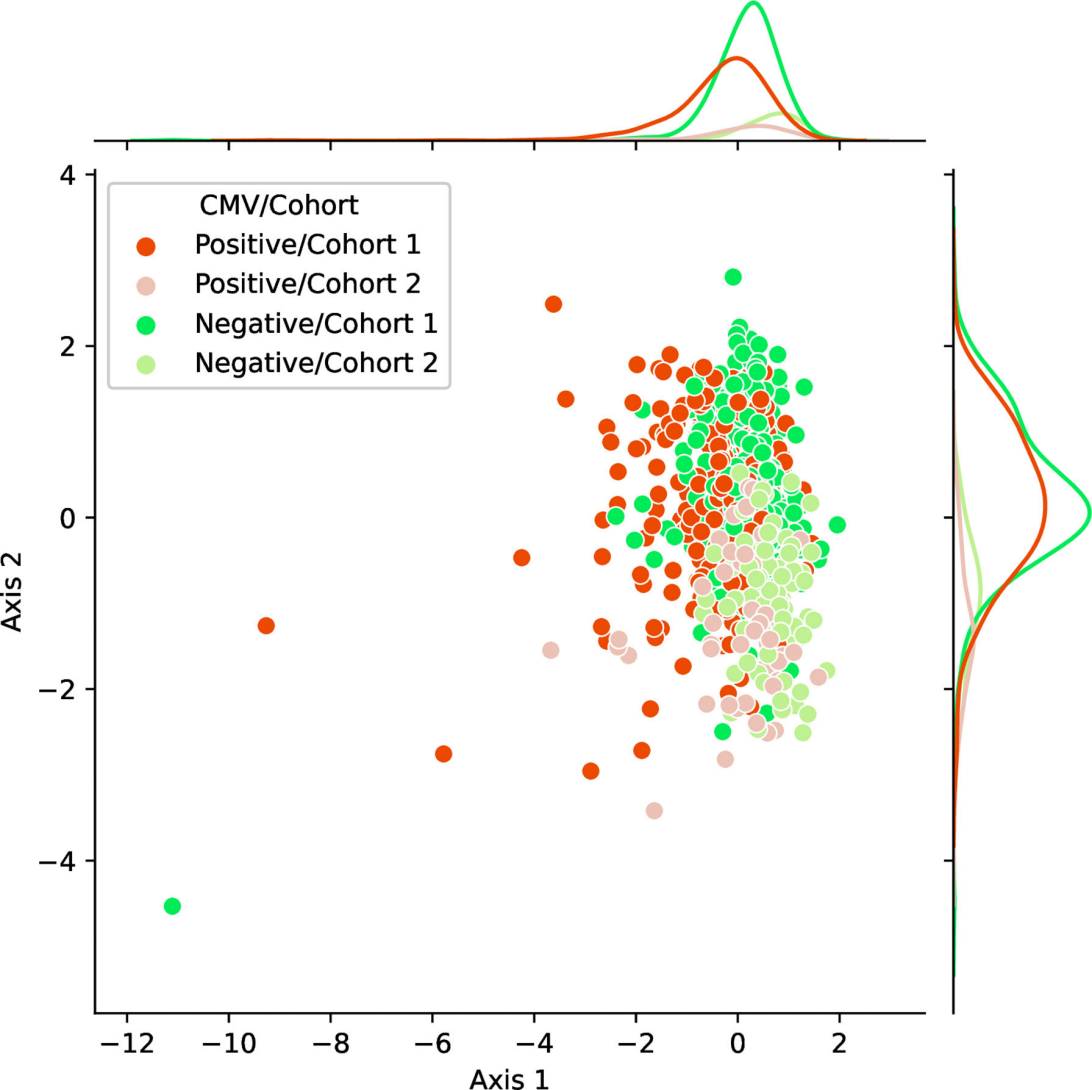
A scatter plot of samples in the Emerson dataset shown in the two-dimensional latent space of the 3-mer feature vectors (9,261 dimensions) obtained by an unsupervised learning method, GPLVM. Axis 1 and 2 correspond to the two principal axes of the latent space learned by GPLVM. Each point represents a sample color-coded by its CMV infection status and by the label whether it belongs to either Cohort 1 or 2. The probability distributions shown on each axis represent the projections of data points of each class onto each axis.

We observed the stability and data efficiency of MotifBoost. However, their source is still elusive. One possibility is that the *k*-mer representation itself is already a good feature for the repertoire classification task. To investigate the feature space of MotifBoost, we employed Gaussian Process Latent Variable Model (GPLVM) (52), an unsupervised dimensionality reduction method, to visualize the *k*-mer feature vectors of the Emerson dataset in the two-dimensional space (**Figure 4**). GPLVM is a kind of PCA extended with a probabilistic model and kernel method to deal with non-linear correlations. Therefore, the two axes in the figure are similar to the principal components in PCA. We found that it is possible to classify repertoires by the Cohort and by the CMV infection status using only the *k*-mer features without any supervised learning (**Table 5**).

**Table 5:** The correlation between the features of samples (CMV infection status and Cohort) and the two axes of **Figure 4** measured by the ROC-AUC score.

	Axis 1	Axis 2
CMV Classification	**0.68**	0.53
Cohort Classification	**0.70**	**0.87**

To compute the scores, each axis is treated as a binary classifier by the following procedures: 1) Each point representing a sample is projected onto the axis. 2) The projected coordinate on the axis is used as the prediction for the sample. ROC-AUC scores of such linear classifiers derived by the axes are shown. The bold ROC-AUC scores are significant (p < 0.05) in the sense of Spearman correlation coefficient. Note that the ROC-AUC scores were calculated using both Cohort 1 and 2 to show the separability of the k-mer latent space for both conditions of the CMV and Cohort classification. Therefore, these ROC-AUC scores cannot be directly compared with those of other Figures and Tables in which the scores are computed only using Cohort 2 after training on Cohort1. See **Table 6** for the comparable score.

**Table 6:** The best ROC-AUC score of the CMV classification task by a linear classifier on the two-dimensional latent space of **Figure 4**.

	ROC-AUC	Optimized axis
CMV Classification	0.75	y = 0.25*x*

The best classifier is chosen by the following numerical optimization procedures: 1) Calculate the ROC-AUC scores for various values of slopes of the axis by following the same procedure as **Table 5** but using only the Cohort 1 dataset. 2) Select the best slope that yields the best ROC-AUC score for the Cohort 1 dataset. After the optimization, the ROC-AUC score shown in the table was calculated by using only the Cohort 2 dataset to follow the same train/test split setup as in other Figures and Tables. x and y in the equation shown in the table denote the coordinate on each of Axis 1 and 2 respectively.

In **Figure 4**, the infection status of CMV was correlated mainly with Axis 1, whereas the Cohort was correlated moderately with Axis 2 and weakly with Axis 1. We also found that the ROC-AUC score of 0.75 for CMV classification could be achieved by a linear separation on the dimensionality-reduced *k*-mer feature space (**Tables 5**, **6**). These results indicate that various information, at least being relevant to the repertoire classification task, is appropriately embedded and represented in the *k*-mer-based features. Thus, the stability of MotifBoost may be attributed to the effectiveness of *k*-mer representation of a repertoire.

### Ablation Study of MotifBoost

We observed desirable properties of *k*-mer representation. However, MotifBoost also consists of other components such as GBDT, hyperparameter search (HS), and data augmentation (DA). To investigate the contributions of these components, we conducted an ablation study (**Table 7**). We compared the ROC-AUC scores of multiple experimental conditions in which one or more of the three components (GBDT, HS, DA) were removed or modified. When *N* is small, vanilla GBDT models can perform only as well as Logistic Regression (LR) models. Being combined with both HS and DA, the performance is uplifted. On the other hand, when *N* is large, the choice of GBDT plays a dominant role in the performance. The result suggests that all three components actually contribute to the performance in different ways.

**Table 7:** Ablation study of MotifBoost. The mean ROC-AUC scores of ablated models for the small and large datasets are shown.

Conditions			ROC-AUC		
(lr)1-3 (lr)4-5 Classifier	HS	DA	*N* = 25	*N =*640	
GBDT	+	+	0.71	0.78	Original MotifBoost
GBDT		+	0.63	0.77	Hyperparameter Search (HS) is removed
GBDT	+		0.65	0.78	Data Augmentation (DA) is removed
GBDT			0.50	0.77	Both DA and HS are removed
LR	n.a.	+	0.55	0.64	GBDT is replaced with Logistic Regression (LR)

The performance of ablated models is measured five times by the same setup as **Figure 2** (N = 25, N = 640). The performance of the original MotifBoost is taken from **Figure 2**. For Logistic Regression (LR) classifier, hyperparameter search (HS) is not applicable.

### Analysis of the Latent Information Employed by Each Method

**Figure 5:**
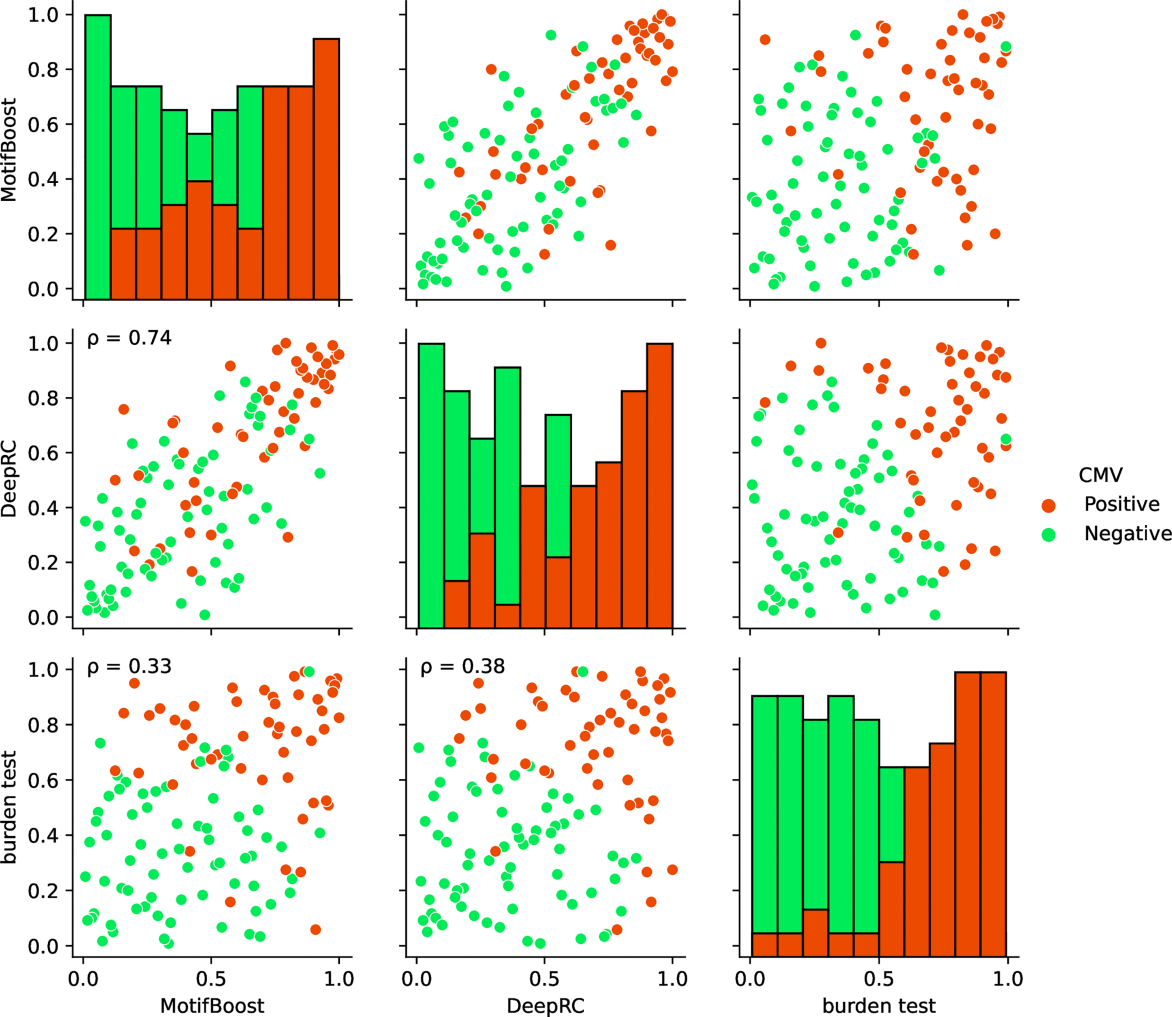
Visualization of the correlations of the prediction profiles between the three methods trained on the full training dataset. In the off-diagonal plots, each axis represents the normalized rank of prediction scores of all test samples (Cohort 2) by the designated method. If a sample is located at 1.0/0.0 on an axis, the method corresponding to the axis gives the sample the highest/lowest prediction score of CMV positiveness. The color of each point indicates the CMV status of that point: positive (red) or negative (green). The *p* on the panels indicates the Spearman correlation. All correlations were significant (*p* < 0.05). Each diagonal plot shows the histograms of the normalized predicted score for CMV positive (red) and negative (green) samples obtained by each method.

Finally, we compared the prediction profiles of the three methods to examine the similarities and differences in the latent information used by the methods. The profiles in **Figure 5** show that the predictions by MotifBoost and DeepRC are similar (*p*=0.74), whereas that of the burden test differs from the others (*p*=0.33 and *p*=0.38).

The similar prediction profiles and ROC-AUC scores of MotifBoost and DeepRC suggest that the two methods employ similar information despite the differences of the underlying algorithms. DeepRC might be learning the features from scratch that contain similar information to the *k*-mer features, and its failure might be related to the collapse of the representation learning at the critical sample size.

On the other hand, the prediction profile of the burden test deviates from those of MotifBoost and DeepRC: some samples are successfully predicted only by the burden test, while others are successfully predicted only by MotifBoost or DeepRC. Moreover, the correlation between the burden test and either MotifBoost or DeepRC is lower than that between MotifBoost and DeepRC. This is remarkable because the profiles were created at *N*=640 where the ROC-AUC score of DeepRC and MotifBoost (around 0.8) is lower than that of the burden test (around 0.89). The correlation between two prediction profiles with high ROC-AUC tends to be high. Nonetheless, the burden test has lower correlations with the other two. This result implies that the burden test exploits different information from MotifBoost or DeepRC at least partially and that the gap between their average performances at *N*=640 might stem from this difference. Further improvement, which balances the best of all the methods, may be possible by scrutinizing such differences in the exploited latent information rather than just by focusing on their performance scores alone.

## Discussion

In this study, we have systematically investigated the performance of the repertoire classification methods with different principles, by focusing on the impact of the dataset size. We evaluated three methods: the burden test comprehensively tests the significance of each sequence based on its frequency in CMV positive and negative samples and uses only the significant sequences as features for classification; DeepRC uses a Transformer-like deep learning architecture to learn both relevant features and classification from data; MotifBoost proposed in this work employs the *k*-mer feature representation and GBDT for classification. We also compared MotifBoost and two *k*-mer based methods (*k*-mer/SMV and *k*-mer/MIL), each of which combines *k*-mer representation with different classification algorithm, i.e., GBDT, SVM, and linear regression model, respectively.

We found that the burden test and DeepRC can suffer from learning instability and the resultant sudden performance degradation when the number of samples drops below a certain critical size. In contrast, MotifBoost not only performs as well as DeepRC on average when trained on a large dataset, but also achieves stable learning with small performance degradation even when being trained on a small dataset. Moreover, MotifBoost performed better than the other two *k*-mer methods. Across all the four additional small datasets, MotifBoost consistently shows superior performance to the other methods. Moreover, MotifBoost can sustain its performance even if each sample contains only a much smaller number of sequences.

### MotifBoost is Useful as a First Step in Tackling the Repertoire Classification Problem

In academic research of repertoires, as discussed in Introduction, datasets with less than 100 samples account for 88% of all datasets. Moreover, the number of sequences in a sample is sometimes severely limited. For example, that of Huth et al. (48) is 1.7 x 10^3^. MotifBoost can work stably and efficiently even under this small to medium sample size conditions. Therefore, MotifBoost is more versatile and applicable to a wider range of problems than the burden test and DeepRC.

In the Emerson dataset, the burden test outperforms DeepRC and MotifBoost in the repertoire classification task if being fed with all the 640 samples for training. However, the sufficient number of samples for training may depend strongly on the difficulty of the classification task and on the quality of the data, which is not easy to estimate in advance when designing an experiment. In addition, as shown in **Figure 3**, the performance of the data-expensive methods is highly volatile if sufficient data is not supplied. Therefore, it is risky to rely only on these unstable methods for practical use.

On the other hand, the performance and variance of MotifBoost depend weakly on data size even if it is lower than 100. Moreover, this performance stability is validated on the four different datasets (**Table 5**). Therefore, it is always beneficial to use MotifBoost together with the data-expensive ones to avoid the case that we fail to detect the potential information in repertoires due to failure of learning.

A stable method like MotifBoost is also preferable from the viewpoint of reproducibility because the performance is relatively steady even if the sample size of the datasets is changed. The other methods, especially the burden test, have a larger variance in the performance. For example, the ROC-AUC score spans from below 0.5 to around 0.8 at *N*=400 in **Figure 2**. Note that any of the two subsampled datasets at *N*=400 share at least 160 samples, because both are subsampled from the full training dataset (640 samples). Even trained on such similar datasets, the performance of the burden method varies greatly. This implies that, if the samples are obtained independently by another experiment to reproduce the reported result, the performance could vary even more. In addition, MotifBoost does not require high-end hardware. Even for the full training dataset of *N*=640, the computation takes about 3 hours on a consumer CPU (Core i7 8700) with about 50GB RAM. MotifBoost balances prediction performance and computation cost.

In conclusion, our proposed MotifBoost can be used as a complementary method to the data-expensive ones for the repertoire classification task because of the following three points: 1) high performance on the small samples; 2) low variance in results and high reproducibility; 3) low hardware requirements. We released a library on GitHub to apply this method easily on the existing RepSeq data formats (https://github.com/hmirin/MotifBoost). Our implementation will be a drop-in replacement for the implementation of the other methods.

### Potential Information Encoded in Repertoire and Its Representations

We also showed that the feature extraction by *k*-mer and unsupervised learning alone can separate CMV infection status to some extent. This suggests that the *k*-mer representation has suitable properties for extracting important features of repertoires.

Even though deep learning methods trained on large-scale datasets attract a surge of interest these days, as demonstrated in this work, they do not necessarily replace the conventional ones developed based on biological domain knowledge. If a relevant data representation like *k*-mer features is known beforehand, there is little need to acquire a similar representation through representation learning. One possible explanation of the performance discrepancy on small datasets between MotifBoost and DeepRC is that DeepRC must perform an extra step of learning the (*k*-mer like) representation, which fails at a small data size.

On the other hand, the existence of a performance gap between the burden test and the others trained on a sufficiently large dataset (640 samples) indicates that the full-length sequence identity, which is utilized only in the burden test, has some special information, which neither DeepRC nor MotifBoost could capture. This possibility is also supported by the fact that the burden test alone succeeded in classification for some samples. However, at the same time, there are also other samples that the burden test could not correctly classify while the others could. Therefore, these methods may focus on, at least partially, different latent information of the repertoires.

The next computational challenge in the repertoire classification task would be the integration of the full-length sequence identity information and the sequence motif information to improve and balance the performance on large datasets and the stability on small ones. Such an attempt would also deepen our biological understanding of how various immunological information is encoded in repertoires.

## Data Availability Statement

All the data analyzed in this paper have been previously published and can be accessed from the original publications. The code for reproducing the results of this paper is available at https://github.com/hmirin/motifboost-paper. A Python library that implements MotifBoost is released at https://github.com/hmirin/MotifBoost.

## Author Contributions

YK and TK: performed study conception and design, and wrote paper; YK: performed the computational analysis. All authors contributed to the article and approved the submitted version.

## Funding

This research is supported by JST CREST JPMJCR2011 and by JSPS KAKENHI Grant Numbers 19H05799.

## Conflict of Interest

The authors declare that the research was conducted in the absence of any commercial or financial relationships that could be construed as a potential conflict of interest.

## Publisher’s Note

All claims expressed in this article are solely those of the authors and do not necessarily represent those of their affiliated organizations, or those of the publisher, the editors and the reviewers. Any product that may be evaluated in this article, or claim that may be made by its manufacturer, is not guaranteed or endorsed by the publisher.
