# Supplementary material for: Comparative Study of Repertoire Classification Methods Reveals Data Efficiency of k -mer Feature Extraction
Source: Front Immunol. 2022 Jul 20;13:797640. doi: 10.3389/fimmu.2022.797640 (PMC9346074; doi:10.3389/fimmu.2022.797640)
Supplement: Supplementary file 1 [file DataSheet_1.pdf]

Table S1 List of publicly available repertoire sequence datasets from TCRdb (26)

Project ID	Project Name	No. of Samples
immunoSEQ90	A large-scale database of T-cell receptor beta (TCRb) sequences and binding associations from natural and synthetic exposure to SARS-CoV-2	1411
immunoSEQ24	Comprehensive T cell repertoire characterization of localized non-small cell lung cancer	569
PRJNA544699	TCR sequencing in NSCLC (TRACERx)	351
PRJEB33490	T cell receptor next-generation sequencing reveals cancer-associated repertoire metrics and reconstitution after chemotherapy in patients with hematological and solid tumors	325
immunoSEQ91	Memory CD4+ T cell receptor repertoire data mining as a tool for identifying cytomegalovirus serostatus	258
immunoSEQ46	Association of Tumor Microenvironment T-Cell Repertoire and Mutational Load With Clinical Outcome After Sequential Checkpoint Blockade in Melanoma	214
immunoSEQ36	T cell clonal expansions in ileal Crohn's disease are associated with smoking behaviour and postoperative recurrence	199
immunoSEQ23	Molecular analysis of primary melanoma T cells identifies patients at risk for metastatic recurrence	199
immunoSEQ02	Immune awakening revealed by peripheral T cell dynamics after one cycle of immunotherapy	160
PRJNA301507	Profiling of Human T-Cell Receptor Repertoire	157
PRJNA493983	Precise tracking of vaccine-responding T-cell clones reveals convergent and personalized response in identical twins	128
immunoSEQ85	Deep Sequencing of T-Cell Receptor DNA as a biomarker of clonally expanded TILs in breast cancer after immunotherapy	115
immunoSEQ83	Neutrophils dominate the immune cell composition in non-small cell lung cancer	114
immunoSEQ84	TCRB technical replicates of PBMC from four donors	107
immunoSEQ80	Successive annual influenza vaccination induces a recurrent oligoclonotypic memory response in circulating T follicular helper cells	102

PRJNA495603	Comprehensive Analysis of TCR-β Repertoire in Patients with Neurological Immune-mediated Disorders (human)	100
PRJNA312319	Tracking T-cell immune reconstitution after TCRαβ/CD19-depleted hematopoietic cells transplantation in children	98
PRJNA516296	Homo sapiens TCR-seq Raw sequence reads on Graves' ophthalmopathy disease	96
immunoSEQ77	Somatic mutations in clonally expanded cytotoxic T lymphocytes in patients with newly diagnosed rheumatoid arthritis	94
immunoSEQ79	The T-cell receptor repertoire influences the tumor microenvironment and is associated with survival in aggressive B-cell lymphoma	92
immunoSEQ67	T-cell receptor β chains show abnormal shortening, repertoire diversity and sharing in type 1 diabetes	88
immunoSEQ18	T cell receptor sequencing-based assay identifies cross-reactive recall CD8+ T cell clonotypes against autologous HIV-1 epitope variants	84
immunoSEQ31	Deletion of donor-reactive T cell clones following human liver transplantation	84
PRJNA579190	T-cell repertoire analysis of Susac Syndrome patients	83
PRJNA393498	TCR repertoire of ankylosing spondylitis patients	78
PRJNA473147	Antigen-specific T-cell receptor signatures of cytomegalovirus infection (human)	75
PRJNA602827	Alternative splicing and the epigenome in CML remission [RNA-Seq] (human)	75
immunoSEQ45	Subclonal STAT3 Mutations Solidify Clonal Dominance	73
PRJNA491656	T-cell receptor repertoire analysis of advanced solid tumors in first-in-human phase 1 study of IT1208, a defucosylated humanized anti-CD4 depleting antibody [TCR-seq] (human)	72
immunoSEQ01	T-cell Repertoire in Combination with T-cell Density Predicts Clinical Outcomes in Patients with Merkel Cell Carcinoma	72
immunoSEQ52	Radiotherapy induces responses of lung cancer to CTLA-4 blockade	72
PRJNA273698	Homo sapiens Targeted Locus (Loci)	71
PRJEB38339	Next Generation Sequencing of T and B cell receptor repertoires from COVID-19 patients showed signatures associated with severity of disease	69

immunoSEQ20	A phase Ib study of preoperative, locoregional IRX-2 cytokine immunotherapy to prime immune responses in patients with early stage breast cancer	63
PRJNA377207	Single cell and bulk TCR sequences - gluten challenge study	61
immunoSEQ74	Contribution of systemic and somatic factors to clinical response and resistance to PD-L1 blockade in urothelial cancer: An exploratory multi-omic analysis	60
PRJNA506151	Heterogeneous Tumor Microenvironment of Non-Small Cell Lung Cancer	57
immunoSEQ47	Multifactorial Heterogeneity of Virus-specific T Cells and Association with the Progression of Human Chronic Hepatitis B Infection	56
PRJNA329041	TCR repertoire of identical twins study	51
PRJNA258001	T-cell receptor repertoires in HIV-infected patients and healthy controls	50
immunoSEQ55	CD4+ and CD8+ autoreactive T cells in narcolepsy patients target self-antigens of hypocretin-producing neurons	49
immunoSEQ66	T-cell receptor sequencing of early stage breast cancer tumors identifies altered clonal structure of the T-cell repertoire	49
PRJNA330606	T cell repertoire sequencing for tumor,non-tumor and lymph node tissues of breast cancer patients	48
PRJNA577794	Comprehensive analysis of antiviral adaptive immunity formation and reactivation down to single cell level	48
PRJNA436233	Homo sapiens Raw sequence reads	48
PRJNA316033	Homo sapiens Raw sequence reads	45
PRJNA516984	retroperitoneal liposarcoma TCR β CDR3 sequencing	45
immunoSEQ43	Mobilization of CD8+ T cells via CXCR4 blockade facilitates PD-1 checkpoint therapy in human pancreatic cancer	45
PRJNA391483	Shared αβ T Cell Receptor Usage in Lungs of Sarcoidosis Patients with Löfgren's Syndrome	44
immunoSEQ70	Abnormalities of T cell receptor repertoire in CD4+ regulatory and conventional T cells in patients with RAG mutations: implications for autoimmunity.	43
PRJNA325496	Homo sapiens Targeted Locus (Loci)	42

PRJNA395098	Deep sequencing and flow cytometric characterization of expanded effector memory CD8+CD57+ T cells frequently reveals T-cell receptor Vß oligoclonality and CDR3 homology in acquired aplastic anemia	42
PRJNA79707	Exhaustive T-cell repertoire sequencing of human peripheral blood samples reveals signatures of antigen selection and a directly measured repertoire size of at least 1 million clonotypes	42
immunoSEQ12	Immuno-genomic landscape of osteosarcoma	41
PRJNA300878	Homo sapiens B and T cell repertoire - MZ twins	40
immunoSEQ63	Contraction of T cell richness in lung cancer brain metastases	40
PRJNA427746	Immune Repertoire Sequencing Using Molecular Identifiers Enables Accurate Clonality Discovery and Clone Size Quantification	39
immunoSEQ59	Patients with CD3G mutations reveal a role for human CD3g in Treg diversity and suppressive function	38
immunoSEQ34	T cell repertoire remodelling following post-transplant T cell therapy coincides with clinical response	38
immunoSEQ37	Clonal replacement of tumor-specific T cells following PD-1 blockade	37
PRJNA356992	Homo sapiens Transcriptome or Gene expression	35
immunoSEQ75	Mismatch repair deficiency predicts response of solid tumors to PD-1 blockade	35
immunoSEQ68	Memory CD4+ T cell receptor repertoire data mining as a tool for identifying cytomegalovirus serostatus	35
PRJNA318421	Homo sapiens TCR repertoire of CD8 T lymphocytes against CMV-pp65 NLV and Flu-M1 GIL	34
immunoSEQ57	Co-expression of CD39 and CD103 identifies tumor-reactive CD8 tumor-infiltrating lymphocytes in human solid tumors	33
immunoSEQ04	TCR repertoire sequencing identifies synovial Treg cell clonotypes in the bloodstream during active inflammation in human arthritis	32
PRJNA315543	The Glioma-Infiltrating T Cell Receptor Repertoire	31
PRJNA422601	Characterization of distinct T cell receptor repertoires in tumor and adjacent non-tumor tissues from lung cancer patients	30
immunoSEQ82	CD49a Expression Defines Tissue-Resident CD8+ T Cells Poised for Cytotoxic Function in Human Skin	30

immunoSEQ21	Model to improve specificity for identification of clinically-relevant expanded T cells in peripheral blood	29
PRJNA321261	Identification of shared TCR sequences from T cells in human breast cancer using emulsion RT-PCR	28
immunoSEQ25	High PD-L1/CD86 MFI ratio and IL-10 secretion characterize human regulatory dendritic cells generated for clinical testing in organ transplantation	28
immunoSEQ44	Altered T Cell Receptor Beta Repertoire Patterns in Pediatric Ulcerative Colitis	26
immunoSEQ08	Maternal T cells in the Human Placental Villi Support an Allograft Response During Non-Infectious Villitis	25
PRJNA390125	TCR repertoire sequencing of T cell subsets from healthy individuals	24
PRJNA412739	MAIT cell TCR repertoire during Salmonella Paratyphi A infection	24
immunoSEQ71	Tumor-infiltrating Merkel cell polyomavirus-specific T cells are diverse and associated with improved patient survival	24
PRJNA385561	Immune reconstitution of systemic sclerosis patients after autologous hematopoietic stem cell transplantation	23
PRJNA509233	TCRbeta rearrangements in T-ALL	23
PRJNA393071	Minor changes of T cell receptor repertoire in response to subunit inactivated influenza vaccine	23
PRJNA389805	T cell receptor repertoire in blood and skin of healthy volunteers with immunological memory to mycobacteria	20
PRJNA477518	T cell receptor repertoire sequencing of healthy individuals	20
immunoSEQ40	AIRE expression controls the peripheral selection of autoreactive B cells	20
immunoSEQ69	Public clonotypes and convergent recombination characterize the naïve CD8+ T-cell receptor (TCR) repertoire of extremely preterm neonates.	19
immunoSEQ03	A circulating reservoir of pathogenic-like CD4+ T cells shares a genetic and phenotypic signature with the inflamed synovial micro-environment	18
immunoSEQ22	Distinct immune characteristics distinguish hereditary and idiopathic chronic pancreatitis	18

PRJNA297261	TCR β-chain repertoire characterisation of regulatory and conventional T cells in peripheral blood from breast cancer patients and healthy individuals	17
immunoSEQ72	T-cell localization, activation and clonal expansion in human pancreatic ductal adenocarcinoma	16
immunoSEQ06	Alloreactive T Cell Receptor Diversity against Structurally Similar or Dissimilar HLA-DP Antigens Assessed by Deep Sequencing	15
immunoSEQ62	Alloreactive T Cell Receptor Diversity against Structurally Similar or Dissimilar HLA-DP Antigens Assessed by Deep Sequencing	15
PRJNA280417	Multiple sclerosis T-cell Receptor Beta Chain sequences	14
PRJNA510967	Bone marrow central memory and memory stem T-cell exhaustion in AML patients relapsing after HSCT	14
immunoSEQ26	Endogenous CD4+ T cells recognize neoantigens in lung cancer patients, including recurrent oncogenic KRAS and ERBB2 (Her2) driver mutations.	14
PRJNA449605	TCR repertoire sequencing on Graves' ophthalmopathy disease	13
PRJNA376866	Single cell and bulk TCR sequences - longitudinal study	12
immunoSEQ39	Human urothelial bladder cancer generates a clonal immune response: The results of T-cell receptor sequencing	12
immunoSEQ53	High prevalence of S. pyogenes Cas9-reactive T cells within the adult human population	12
immunoSEQ56	Memory B Cells Activate Brain-Homing, Autoreactive CD4+ T Cells in Multiple Sclerosis	12
PRJNA325416	Heterogeneity of tumor-infiltration lymphocytes ascribed to local immune status instead of neoantigens by multi-reginal omics analysis of glioblastoma multiforme	11
PRJNA494572	Sequencing the Peripheral Blood B and T cell Repertoire - Quantifying robustness and limitations	11
PRJNA374973	Generation of mature T cells from human hematopoietic stem/progenitor cells in artificial thymic organoids	11
immunoSEQ27	Lack of specific T- and B-cell clonal expansions in multiple sclerosis patients with progressive multifocal leukoencephalopathy	11

PRJNA79519	High throughput sequencing reveals a complex pattern of dynamic interrelationships among human T cell subsets	10
PRJNA633317	Longitudinal high-throughput TCR repertoire profiling reveals the dynamics of T cell memory formation after mild COVID-19 infection	10
PRJNA429872	Homo sapiens Raw sequence reads	10
immunoSEQ19	Epstein-Barr Virus Epitope–Major Histocompatibility Complex Interaction Combined with Convergent Recombination Drives Selection of Diverse T Cell Receptor alpha and beta Repertoires	10
immunoSEQ64	T cell phenotype and T cell receptor repertoire in untreated patients with major depressive disorder	10
immunoSEQ30	Clonal and Constricted T cell repertoire in Common Variable Immune Deficiency	9
PRJNA450033	TCR repertoire of effector CCR7-CD38+ cells accumulating during experimental infection with Typhoidal Salmonella (human)	8
PRJNA214848	Distinctive properties of identical twins' TCR repertoires revealed by high-throughput sequencing	8
PRJNA229070	Homo sapiens Targeted Locus (Loci)	7
PRJNA544470	T cell receptor sequencing	7
PRJNA389678	Quantifying T cell and TCR response using KT2 cells	7
PRJNA318495	Human T cell receptor sequencing	6
immunoSEQ09	Intratumoral heterogeneity and clonal evolution in liver cancer	6
immunoSEQ78	Broad TCR repertoire and diverse structural solutions for recognition of an immunodominant CD8+ T cell epitope	6
immunoSEQ05	Comprehensive Characterization of a Next-Generation Antiviral T-Cell Product and Feasibility for Application in Immunosuppressed Transplant Patients	6
PRJNA577520	The diversity of TCR β chain CDR3 repertoire in patients with severe acne	4
PRJNA517192	High-throughput TCRβ Gene Sequencing	4
PRJNA377124	Epitope specific paired TCR repertoire sequencing	3
immunoSEQ11	Somatic Mutations in Clonally Expanded T-lymphocytes in Patients with Chronic Graft-Versus-Host Disease	3

immunoSEQ65	Hepatitis E virus-induced primary cutaneous CD30+ T cell lymphoproliferative disorder	3
immunoSEQ32	Human CD4+ T cells specific for Merkel cell polyomavirus localize to Merkel cell carcinomas and target a required oncogenic domain	3
PRJNA203441	Profiling the T-cell receptor repertoire of patient with pleural tuberculosis by high through-put sequencing	2
PRJNA208615	Homo sapiens TCR transcriptome	2
immunoSEQ76	Absence of functional fetal regulatory T cells in humans causes in utero organ-specific autoimmunity	2
PRJNA312766	T cell receptor repertoire of Human T1D, T2D patients and controls	1
PRJNA298417	NGS data of T-cell receptor repertoire of naive CD4 and CD8 T-cells in healthy donors	1
PRJNA267461	Repertoire Sequencing of Gastric Tumor-Infiltrated T Lymphocyte	1
immunoSEQ10	Prevalent and diverse intratumoral oncoprotein-specific CD8 T cells within polyoma virus-driven Merkel cell carcinomas	1

Table S2 Parameters searched for the burden test

Parameter Name	
Significance Threshold for the Fisher Exact Test	0.5, 0.05, 0.005, 0.0005, 0.00005
Update Rate for Newton's Method	0.01, 0.001, 0.0001
Number of iterations for Newton's Method	100, 1000, 10000

Parameter Evaluation
ROC-AUC score (5-fold cross-validation)
